# Examining the Relationship Between Cyberchondria and Health Anxiety in Clinical Medical Students

**DOI:** 10.1192/j.eurpsy.2025.1013

**Published:** 2025-08-26

**Authors:** N. Boussaid, R. Feki, S. Omri, I. Ben Ayed, I. Gassara, N. Charfi, J. Ben Thabet, M. Maalej, M. Maalej, N. Smaoui, L. Zouari

**Affiliations:** 1 Psychiatry C, Hedi Chaker university hospital center, Sfax, Tunisia

## Abstract

**Introduction:**

Cyberchondria refers to the phenomenon where individuals experience increased health anxiety due to excessive health-related information-seeking on the internet. This behavior can lead to increased worry, symptom misinterpretation, and the belief that one may be suffering from a serious illness. While internet health-seeking is common, it can become maladaptive and contribute to clinically significant anxiety.

**Objectives:**

The aim of this study was to investigate the prevalence and severity of cyberchondria among medical students, and to explore the relationship between online health-seeking behaviors and health anxiety.

**Methods:**

A cross-sectional survey was conducted among clinical medical students using the **Cyberchondria Severity Scale (CSS)** and the **Short Health Anxiety Inventory (HAI-18).** The CSS is a 12-item self-report questionnaire that assesses the frequency of online health searches, the distress caused by those searches, and the misinterpretation of symptoms. The HAI-18 evaluates the frequency and intensity of health-related worries and behaviors over the past six months. Participants were asked to report their online health-searching habits, emotional responses, and overall health anxiety levels.

**Results:**

A total of 169 clinical students participated in the study, with a predominance of females (74%). The mean age of participants was 23 ± 1.5 years. Regarding family medical history, 62.1% of participants reported a familial history of medical conditions, while 26% had a familial history of psychiatric disorders. Additionally, 39.6% of participants reported that their family members had been hospitalized for a serious illness. A personal medical history was reported by 23.7% of participants, and 21.3% had a documented history of psychiatric disorders. Health-related anxiety was observed in 21.9% of participants and was significantly associated with a history of family member hospitalization for a serious illness (p < 0.05). Regarding cyberchondria, 35.5% of participants reported low levels, 43.2% moderate levels, 20.1% high levels, and 1.2% very high levels. High levels of cyberchondria were significantly associated with higher health anxiety scores (p < 0.001).

**Conclusions:**

The findings suggest a moderate to high prevalence of cyberchondria among clinical medical students, with a strong association between higher cyberchondria scores and increased health-related anxiety. Interventions to reduce cyberchondria should focus on managing health anxiety and mitigating the negative impact of online health information

**Disclosure of Interest:**

None Declared

